# 4-Phenylbutyrate Inhibits Tunicamycin-Induced Acute Kidney Injury via CHOP/GADD153 Repression

**DOI:** 10.1371/journal.pone.0084663

**Published:** 2014-01-08

**Authors:** Rachel E. Carlisle, Elise Brimble, Kaitlyn E. Werner, Gaile L. Cruz, Kjetil Ask, Alistair J. Ingram, Jeffrey G. Dickhout

**Affiliations:** 1 Department of Medicine, Division of Nephrology, McMaster University and St. Joseph's Healthcare Hamilton, Hamilton, Canada; 2 Department of Medicine, Division of Respirology, McMaster University and St. Joseph's Healthcare Hamilton, Hamilton, Canada; Institut National de la Santé et de la Recherche Médicale, France

## Abstract

Different forms of acute kidney injury (AKI) have been associated with endoplasmic reticulum (ER) stress; these include AKI caused by acetaminophen, antibiotics, cisplatin, and radiocontrast. Tunicamycin (TM) is a nucleoside antibiotic known to induce ER stress and is a commonly used inducer of AKI. 4-phenylbutyrate (4-PBA) is an FDA approved substance used in children who suffer from urea cycle disorders. 4-PBA acts as an ER stress inhibitor by aiding in protein folding at the molecular level and preventing misfolded protein aggregation. The main objective of this study was to determine if 4-PBA could protect from AKI induced by ER stress, as typified by the TM-model, and what mechanism(s) of 4-PBA's action were responsible for protection. C57BL/6 mice were treated with saline, TM or TM plus 4-PBA. 4-PBA partially protected the anatomic segment most susceptible to damage, the outer medullary stripe, from TM-induced AKI. *In vitro* work showed that 4-PBA protected human proximal tubular cells from apoptosis and TM-induced CHOP expression, an ER stress inducible proapoptotic gene. Further, immunofluorescent staining in the animal model found similar protection by 4-PBA from CHOP nuclear translocation in the tubular epithelium of the medulla. This was accompanied by a reduction in apoptosis and GRP78 expression. CHOP^−/−^ mice were protected from TM-induced AKI. The protective effects of 4-PBA extended to the ultrastructural integrity of proximal tubule cells in the outer medulla. When taken together, these results indicate that 4-PBA acts as an ER stress inhibitor, to partially protect the kidney from TM-induced AKI through the repression of ER stress-induced CHOP expression.

## Introduction

Endoplasmic reticulum (ER) stress is caused by the accumulation of unfolded or misfolded proteins in the ER [1]. The ER chaperone GRP78 binds to the accumulating unfolded proteins after dissociating from ER transmembrane proteins, where it is typically anchored. These anchor proteins then transduce the signals involved in initiating the unfolded protein response (UPR) [2]. Initiation leads to the activation of three main UPR pathways, the inositol requiring enzyme-1 (IRE1) pathway, the activating transcription factor 6 (ATF6) pathway, and the protein kinase-like ER kinase (PERK) pathway [3]. IRE1 can be found in yeast, and therefore represents the first evolved arm of the UPR. Upon activation, a UPR specific transcription factor (HAC1 in fungi, or XBP1 in animals) is cleaved in two specific positions. This results in a spliced transcript that is translated to the active form of XBP1. It is translocated to the nucleus to act as a transcription factor inducing the expression of UPR genes, including the molecular chaperones GRP78 and GRP94. When ATF6 is activated, it is translocated to the Golgi, where site 1 and site 2 proteases remove the luminal domain and the transmembrane anchor, respectively. This allows the N-terminal cytosolic fragment to translocate to the nucleus, activating UPR target genes [4]. PERK is an ER-resident transmembrane kinase, which is autophosphorylated when unfolded or misfolded proteins accumulate in the ER. Phosphorylated PERK phosphorylates eIF2α, which then leads to general protein translation attenuation and the increased transcription of ATF4. The expression of the ATF4 target CHOP, also known as GADD153, is induced, which increases the formation of the GADD34-PP1 complex. This goes on to dephosphorylate phosphorylated eIF2α [4]. This re-initiation of protein synthesis through eIF2α dephosphorylation may be why CHOP exerts proapoptotic effects in cells that have not yet resolved ER stress [5].

GRP78 and CHOP are well-established markers of ER stress and UPR activation. CHOP is a proapoptotic UPR response gene and may play a role in the contribution of ER stress to acute tubular necrosis (ATN) and the resulting acute kidney injury (AKI). AKI, the primary pathology of which is ATN, can be caused by ischemia [6], [7], nephrotoxic drugs [8], [9], or radiocontrast medium [10]. The tubular injury in response to many of these insults has been associated with ER stress [8]–[10]. In addition, tunicamycin (TM), a known inducer of ER stress, has previously been used as a model of antibiotic-induced AKI [11]. It prevents N-linked glycosylation, and has both antibiotic and antiviral properties. Several other studies have shown its effects on the kidney, including upregulated ER stress response proteins, extensive tubular interstitial damage at the cortico-medullary junction, and increased apoptosis [5], [11]–[15].

4-phenylbutyrate (4-PBA) is a low molecular weight chemical chaperone that is currently approved for clinical use in urea cycle disorders. 4-PBA has 3 main biologic effects: it is an ammonia scavenger [16], a weak histone deacetylase (HDAC) inhibitor [17], and an ER stress inhibitor [18]–[20]. It has been shown to restore glucose homeostasis in obese mice [21], and has been used in clinical trials for treatment of cystic fibrosis [22], sickle cell disease [23], neurodegenerative diseases [24] and certain cancers [25]–[27]. Many of theses intervention trials with 4-PBA have relied on the effect of this drug to reduce ER stress.

We therefore hypothesized that 4-PBA would prevent kidney damage in a TM model of AKI through inhibition of ER stress-induced CHOP expression. To investigate this hypothesis, we examined a number of ER stress markers, as well as apoptotic markers both *in vitro* and *in vivo*. This is the first *in vivo* study, to our knowledge, detailing a renoprotective effect of 4-PBA in AKI.

## Methods

### Ethics Statement

All animal work was done in accordance with and approved by the McMaster University Animal Research Ethics Board.

### Cell Culture

HK-2 cells were used as a cell model system of human renal proximal tubular epithelial cells. These cells are an immortalized human proximal tubule cell line obtained from ATCC [28]. HK-2 cells were cultured in a 1∶1 ratio of DMEM 1 g/L glucose media (Invitrogen; Carlsbad, CA) and F12 GlutaMAX nutrient mix (Invitrogen) containing 1× penicillin/streptomycin antibiotic (Invitrogen) and 0.5× non-essential amino acids (Invitrogen). For Western blotting experiments, cells were grown to confluence on 6-well tissue culture plates (BD Falcon, Mississauga, Canada) and for apoptosis experiments, confluent cells were treated on borosilicate glass cover slips to allow subsequent microscopic analysis.

### Reagents

DMSO (Sigma-Aldrich) was used to dissolve non-water soluble reagents and as a vehicle control. TM was purchased from Sigma-Aldrich (St. Louis, MO). 4-PBA sodium salt was purchased from Scandinavian Formulas (Sellersville, PA). Paraformaldehyde was obtained as a 4% solution in phosphate buffered saline (BioLynx Inc; Brockville, Canada) and used for fixation.

### Animal Studies

Wild type C57BL/6 mice were purchased from Charles River (Charles River Laboratories International, Inc., Wilmington, MA) and CHOP^−/−^ mice were obtained from Jackson Laboratories (Stock Number: 005530). Mice were maintained at McMaster University with free access to food and drinking water. Animals were housed with a 12-hour light dark cycle. Wild type animals ranging from 21–28 weeks of age were utilized to examine the effects of 4-PBA on AKI. Sham mice (N = 13) had a mean body weight of 33.2 g. TM-treated mice (N = 14) weighed on average 29.0 g. TM with 4-PBA-treated mice (N = 15) had an average weight of 28.9 g. Mice were given regular drinking water or 4-PBA (1 g/kg/day) in the drinking water for 10 days. On the seventh day, mice were intraperitoneally (I.P.) injected with TM (0.5 mg/kg). On the tenth day, mice were sacrificed, and kidneys were harvested. CHOP^−/−^ mice were utilized from 12–30 weeks of age. Sham mice (N = 4) had an average weight of 20.3 g, while TM-treated mice (N = 6) had a mean weight of 23.2 g. Mice were I.P. injected with TM and three days afterwards were sacrificed via exsanguination, and kidneys were harvested.

### Assessment of Renal Pathology

Kidneys were sectioned and stained with Periodic Acid-Schiff reagent (PAS). Briefly, kidney sections were mounted on microscope slides. Sections (4 µm thick) were cut, air-dried, and deparaffinized through a series of xylene and graded ethanol (100%–70%). Slides were oxidized in 1% aqueous periodic acid, treated with Schiff reagent, counterstained with haematoxylin, and then mounted. The kidneys were scored for tubular damage by two independent investigators blinded to the treatment groups as follows: (0) 0% kidney damage, (1) 1–25% kidney damage, (2) 26–50% kidney damage, (3) >50% kidney damage. Damage was defined as vacuolization of the tubular epithelium, as well as its denudation and loss of cellular nuclei. Scores were collected then averaged to produce a single score for each animal. Kidney sections also underwent Masson's Trichrome staining to further evaluate tissue damage and fibrosis. Nine or more animals were scored in each treatment group.

### Urine and Plasma Analysis

Animals were placed in metabolic cages prior to sacrifice. After 24 h, food and water intake were measured, and urine was collected. Urine was then sent to our in-house laboratory (St Joseph's Healthcare Hamilton) for levels of urinary protein to be measured. Animals were sacrificed via exsanguination; blood was collected from the left ventricle in heparinized tubes, and subsequently centrifuged to separate the plasma. Plasma samples were sent to our in-house laboratory for levels of plasma creatinine to be measured.

### Gel Electrophoresis

Total cell lysates were generated in 4× SDS lysis buffer with protease inhibitor cocktail (complete Mini; Roche; Laval, Canada) and phosphatase inhibitor cocktail added. Protein levels were determined using BioRad DC Protein Assay (BioRad, Mississauga, Canada) for control of protein loading. Cell lysates were subjected to electrophoretic separation in an SDS-PAGE reducing gel (BioRad). Primary antibodies were detected using appropriate horseradish peroxidase-conjugated secondary antibodies and ECL Western Blotting Detection Reagents (GE Healthcare, Mississauga, Canada), as described previously [29]–[31]. CHOP antibody (sc-793, Santa Cruz Biotechnology; Santa Cruz, CA) was diluted 1∶200, KDEL antibody (SPA-827, Stressgen) was diluted 1∶1000, and β-actin antibody (Sigma) was diluted 1∶4000. Results were densitometrically quantified using ImageJ software (NIH, Bethesda, MD, ver. 1.43) and expressed as a ratio of β-actin loading control.

### Apoptosis Assay

A terminal deoxynucleotidyl transferase dUTP nick end labelling (TUNEL) staining kit (TMR-In situ cell death detection kit, Roche) was utilized to label cells undergoing apoptosis *in vitro*, as previously described [32]. Apoptotic cells and total cells were counted and analyzed using ImageJ software. Total cell counts were based on 4′,6-diamidino-2-phenylindole (DAPI) nuclear staining. For cells to be considered TUNEL positive the red emission of the TMR-oligo tag was required to overlap with DAPI nuclear staining. This allowed the number of apoptosis-positive cells to be expressed as a percentage of total cells. Tissue sections were stained for apoptosis using the same TUNEL assay kit according to the manufacturer's instructions. In this case, the level of apoptosis was expressed as the number of apoptotic cells per high-power field.

### Transfections

HK-2 cells were transfected with pcDNA3.1 control vector alone or with the pcDNA3.1 vector containing the open reading frame for the human CHOP gene using FuGENE 6 transfection reagent (Roche) at a 6∶1 ratio. Cells were then fixed with 4% paraformaldehyde, permeabilized, and stained with TUNEL and anti-CHOP antibodies (sc-575; Santa Cruz). HK-2 cells were transfected with CHOP siRNA (Thermo Scientific, Dharmacon ON-TARGET plus, SMARTpool) as per the manufacturer's instructions. Briefly, cells incubated overnight in a 6-well plate in antibiotic-free complete medium. siRNA and DharmaFECT transfection reagent were diluted in serum-free medium. After a five minute incubation, siRNA and DharmaFECT transfection reagent were combined and incubated for a further 20 mins. The transfection medium was then added to each well. Cells incubated at 37°C for 72 h, and were subsequently treated with TM for 48 h. Cells were then fixed with 4% paraformaldehyde and stained for TUNEL. A scrambled siRNA (Dharmacon, Non-targeting siRNA#1) was used as a siRNA transfection control.

### Immunofluorescence

Sections (4 µm thick) were cut, air-dried, and deparaffinized through a series of xylene and graded ethanol (100%–70%). Heat-Induced Epitope Retrieval was performed in Retrieve-All-2 buffer (Signet Laboratories, Dedham, MA) pH 10, for 30 min followed by 0.1% Triton-X for 10 min at room temperature for CHOP staining [12]. No antigen retrieval was used for GRP78 staining [32]. Tissues were stained for CHOP to determine if treatment with TM or 4-PBA resulted in modifications in the expression of this transcription factor. Sections were incubated with either a rabbit anti-CHOP antibody (1∶40, sc-575; Santa Cruz Biotechnology) or a goat anti-GRP78 antibody (1∶40, sc-1050; Santa Cruz Biotechnology). The primary antibody was detected using a species-specific secondary antibody conjugated to an Alexa dye at 647 nm excitation wavelength, producing an emission maximum at 668 nm in the far-red region of the spectrum (1∶500; Invitrogen). This emission wavelength was used due to the high levels of autofluorescence in the renal tubular epithelium at the typical emission wavelengths for FITC (520 nm) or tetramethylrhodamine (580 nm). Sections were incubated with 100 ng/ml DAPI to label cell nuclei and mounted with Permafluor (Thermo Scientific).

### Fluorescence Microscopy

An Olympus IX81 Nipkow scanning disc confocal microscope was used for fluorescence microscopy. Image analysis was performed using Metamorph image analysis software (Molecular Devices, Sunnyvale, CA), as previously [32]. CHOP-positive cell nuclei were counted and expressed as a percent of total cell nuclei. Cell nuclei were identified with DAPI DNA-based staining and quantified using the cell scoring application in Metamorph software (ver 7.71). Utilizing this system, cell nuclei were gated to be between 5 and 20 microns in size and positivity for CHOP was assessed by significantly increased fluorescence intensity over tubular epithelial background utilizing Adaptive background correction™. Apoptotic cells were counted and expressed as the number of TUNEL positive nuclei in each high power field. CHOP analysis consisted of nine animals per group, while TUNEL analysis consisted of six animals per group. Six microscope fields were randomly sampled in both the cortex and the medulla. This allowed the quantification of approximately 5000 cells for CHOP or TUNEL staining per animal per kidney region. GRP78 positive cells were quantified by thresholding for the specific emission of the secondary antibody tag (665 nm), and allowing the software to detect the percent thresholded area for each image, producing the area density of the quantified protein.

### Transmission Electron Microscopy (TEM)

TEM was performed to assess the ultrastructural features of the proximal tubular cells (pars recta) of the outer medulla in wild type sham-, TM-, and TM + 4-PBA-treated mice, as well as CHOP^−/−^ mice with or without TM treatment. Briefly, kidney tissues were fixed in 2% glutaraldehyde in 0.1 M cacodylate buffer, followed by fixation in 1% OsO_4_ in 0.1 M cacodylate buffer. The tissue was then dehydrated in a graded series of ethanol and embedded in Spurr's resin for ultra-thin sectioning. Toluidine blue sections were cut to visualize the outer stripe of the medulla and once localized, were marked to allow the blocks to be trimmed for ultra-thin sectioning in this region. Ultra-thin sections were cut to approximately 100 nm in thickness with an ultramicrotome. Sections were then stained with uranyl acetate and lead citrate and observed with a JEOL 1200EX TEMSCAN (Tokyo, Japan) at 80 KV.

### Statistical Analysis

Quantitative results were expressed as the mean ± SEM and statistically analyzed using the Student's t-test. The significance of differences in the means was assigned at a level of less than a 5% probability (P<0.05) of the difference occurring by chance.

## Results

**Figure 1:**
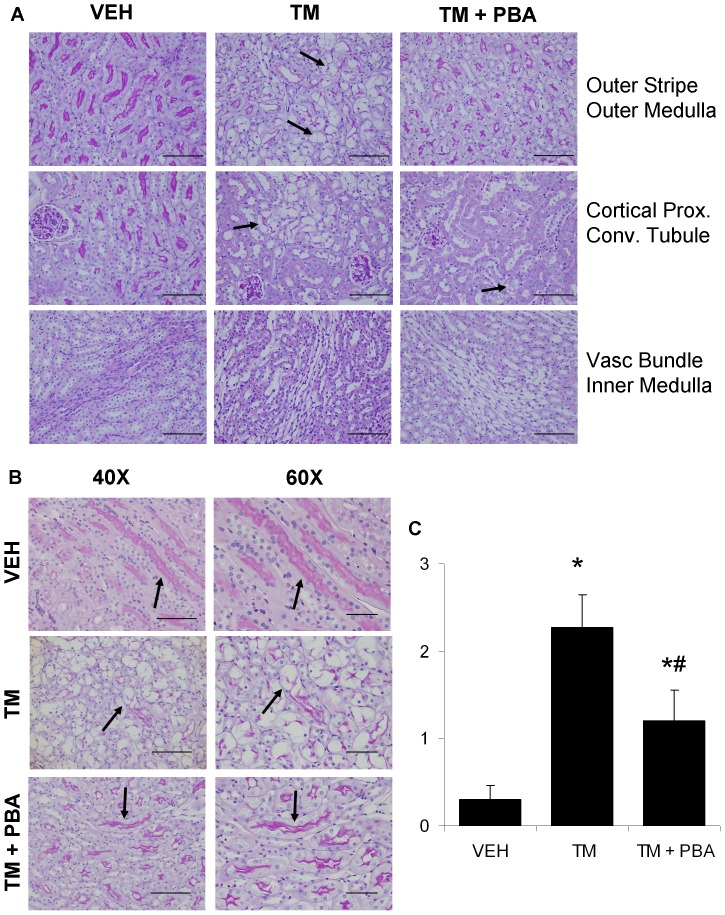
Anatomical structures affected by tunicamycin (TM)-induced acute kidney injury. C57BL/6 wild type mice were treated with saline (VEH), TM for 3 days, or pre-treated with 4-PBA for 7 days followed by 3 days of TM and 4-PBA co-treatment. PAS staining of mouse kidney sections indicate that TM causes acute kidney injury primarily in the outer stripe of the outer medulla (Outer Stripe Outer Medulla, arrows) of the kidney. 4-PBA treatment combined with TM inhibits this effect. TM also induced cortical proximal convoluted tubule (Cortical Prox. Conv. Tubule, arrow) damage; this effect was only partially inhibited by 4-PBA (arrow). Vascular bundles (Vasc Bundle Inner Medulla) were unaffected by TM-induced acute kidney injury at this dose (0.5 mg/kg) or 4-PBA treatment (A; bar = 100 µm). Higher magnification images show the pars recta of the outer medulla in all treatment groups (arrows) and the damage induced by TM and its inhibition by 4-PBA (B; 40×, bar = 100 µm; 60×, bar = 50 µm). The PAS-stained kidneys were scored for tubular damage as follows: (0) 0% kidney damage, (1) 1–25% kidney damage, (2) 26–50% kidney damage, (3) >50% kidney damage. Results indicate that 4-PBA inhibits tubular damage mediated by TM treatment (C). N = 10. *, P<0.05 vs VEH; ^#^, P<0.05 vs TM.

Wild type C57BL/6 mice were randomized into three groups: (1) control (VEH), treated with saline (I.P.; N = 13), (2) treated with TM (0.5 mg/kg, I.P.; N = 14) or (3) co-treated with TM (0.5 mg/kg, I.P.) and 4-PBA (1 g/kg/day, drinking water; N = 15). Mouse kidney sections stained with PAS were examined for pathology in the major anatomical structures of the kidney to determine the precise location of the TM-induced AKI. The outer stripe of the outer medulla was the major region of damage induced by TM injection at this dose. The pars recta were identified as straight segments of proximal tubule, as shown by the presence of a prominent PAS-positive brush border. Damage was characterized by tubular atrophy, loss of brush border and epithelial cell vacuolization in the proximal tubule cells of the pars recta of the outer stripe of the outer medulla (Figure 1A, arrows). These changes were observed less frequently in animals pre-treated with 4-PBA. TM-injected animals also showed damage to the proximal convoluted tubules of the renal cortex with epithelial cell vacuolization (Figure 1A, arrow), as observed previously [12]. Treatment with 4-PBA partially inhibited this injury. The vascular bundles of the renal medulla showed no gross histological change upon TM treatment (Figure 1A). The kidneys were scored as described in the methods. Results show that mice treated with TM alone displayed significantly more damage when compared with saline-treated mice. Mice co-treated with TM and 4-PBA suffered from less kidney damage than TM alone-treated mice, particularly in the pars recta (arrows; Figure 1B and 1C). Substantive proteinuria was not expected in this model at the 0.5 mg/kg dose of TM, however, proteinuria levels were measured and no statistical significance was found between any of the groups. 24 h protein levels were as follows: VEH, 6.23±2.05 mg; TM, 3.13±1.29 mg; TM ±4−PBA, 5.07 + 0.60 mg.

**Figure 2:**
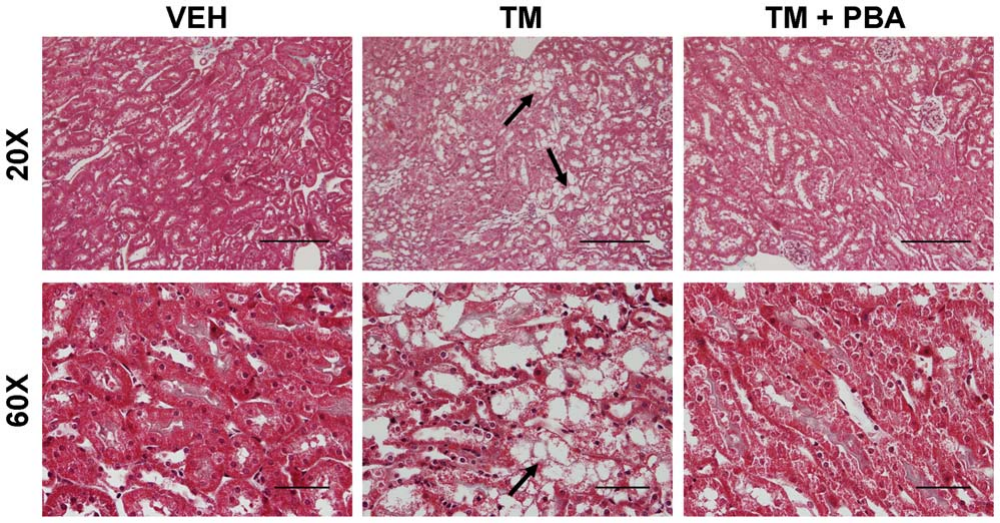
Damage to the outer stripe of the outer medulla is induced by tunicamycin (TM). C57BL/6 wild type mice were treated with saline (VEH), TM for 3 days, or pre-treated with 4-PBA for 7 days followed by 3 days of TM and 4-PBA co-treatment. Masson's Trichrome staining of mouse kidney sections indicate that TM causes acute kidney injury in the outer stripe of the outer medulla (arrows). This damage is prevented by co-treatment with 4-PBA. However, renal interstitial fibrosis was not induced by treatment with TM or 4-PBA (20×, bar = 200 µm; 60×, bar = 50 µm).

To assess if the renal damage involved interstitial fibrosis, mouse kidney sections were stained with Masson's Trichrome. Similar to PAS staining, Masson's Trichrome staining demonstrated that most damage in the kidney occurred in the outer stripe of the outer medulla (Figure 2, arrows). A significant amount of damage occurred in TM-treated mice, but not in TM + 4−PBA−treated mice (Figure 2). Trichrome staining also revealed that TM-induced AKI did not elicit a fibrotic response.

To determine if TM-induced AKI at the 0.5 mg/kg dose resulted in detectable functional damage, creatinine levels were measured. Blood was collected from mice immediately prior to sacrifice. Blood plasma was subsequently analyzed, and it was determined that creatinine levels for TM and TM + 4−PBA treatment groups did not statistically differ from controls. Creatinine concentrations were as follows: VEH, 37.3±1.86 μmol/L; TM-treated, 34.5±2.23 μmol/L; TM + PBA−treated, 40.0±1.25 μmol/L).

**Figure 3:**
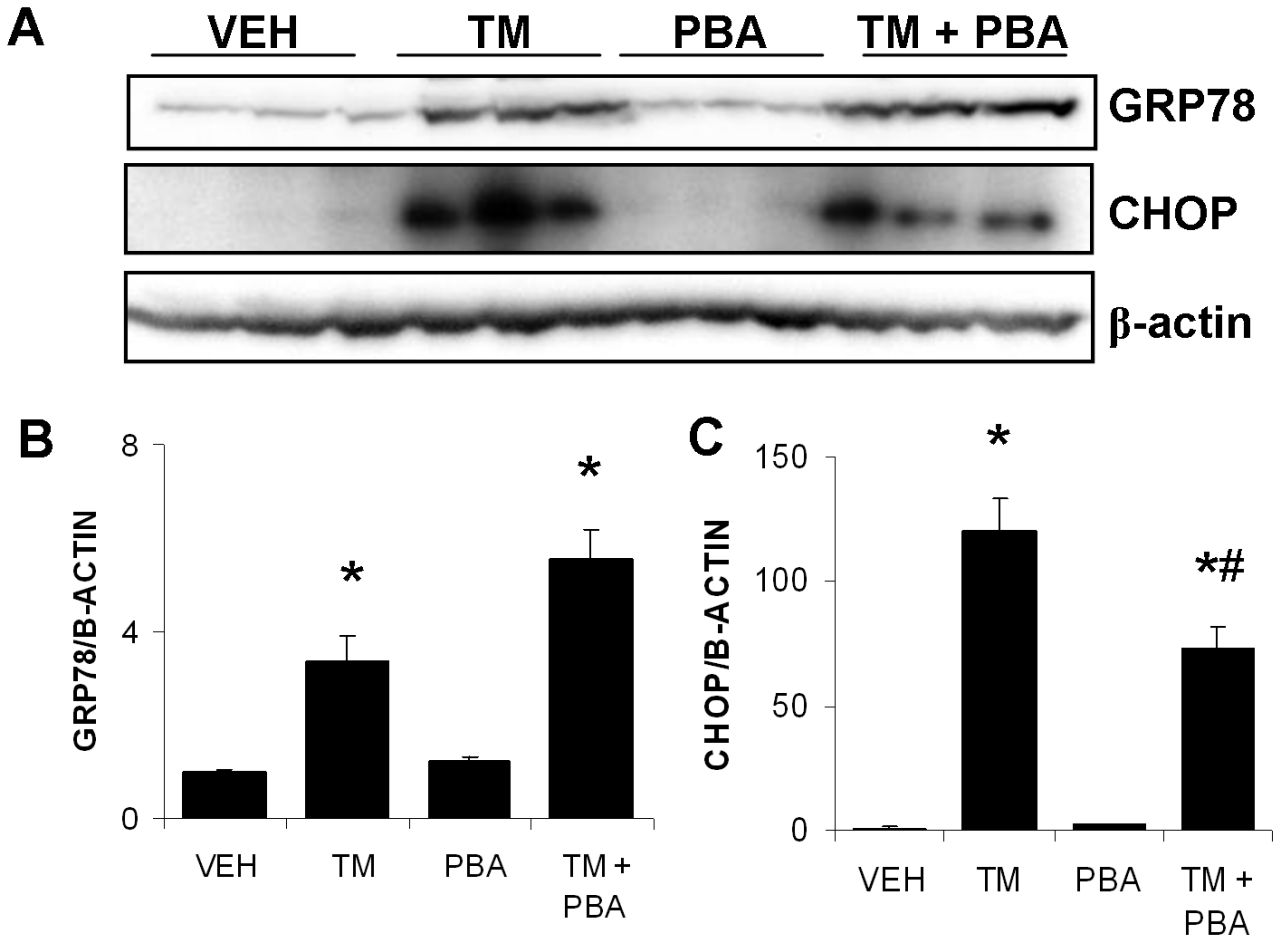
4-PBA significantly inhibits tunicamycin (TM)-induced CHOP expression. HK-2 cells were treated with DMSO (VEH), TM (1 µg/ml), 4-PBA alone (1 mM), or TM with 4-PBA for 24 h. Cells were lysed and underwent Western blotting for GRP78, CHOP and β-actin (A). Densitometric analysis indicates that TM treatment significantly upregulated GRP78 expression. A combined treatment of TM with 4-PBA also resulted in increased GRP78 expression over VEH, but did not show increased expression over TM treatment. 4-PBA alone had no effect on GRP78 expression (B). CHOP expression was increased in response to TM treatment. Co-treatment of TM and 4-PBA resulted in increased CHOP expression, which was significantly higher than VEH treatment, but significantly lower than TM alone treatment. 4-PBA alone-treated cells showed no difference in CHOP expression, when compared with VEH (C). *, P<0.05 vs VEH; ^#^, P<0.05 vs TM.

Since previous work had determined that CHOP^−/−^ mice were protected against TM-induced AKI [5], we attempted to elucidate whether 4-PBA, through its action as an ER stress inhibitor, could repress CHOP expression. In these experiments, HK-2 cells were used as a model of human proximal tubule cells *in vitro*. Cells were treated with VEH, TM, 4-PBA, or TM with 4-PBA for 24 h, and were analyzed via Western blotting (Figure 3A). Tunicamycin treatment increased GRP78 expression, confirming UPR induction. 4-PBA alone did not increase GRP78 expression nor did it repress GRP78 expression when combined with TM treatment (Figure 3B). Densitometric analysis indicated that TM treatment also upregulated CHOP expression in HK-2 cells, while 4-PBA did not. Further, 4-PBA partially prevented TM-induced CHOP expression in human proximal tubular cells (Figure 3C).

**Figure 4:**
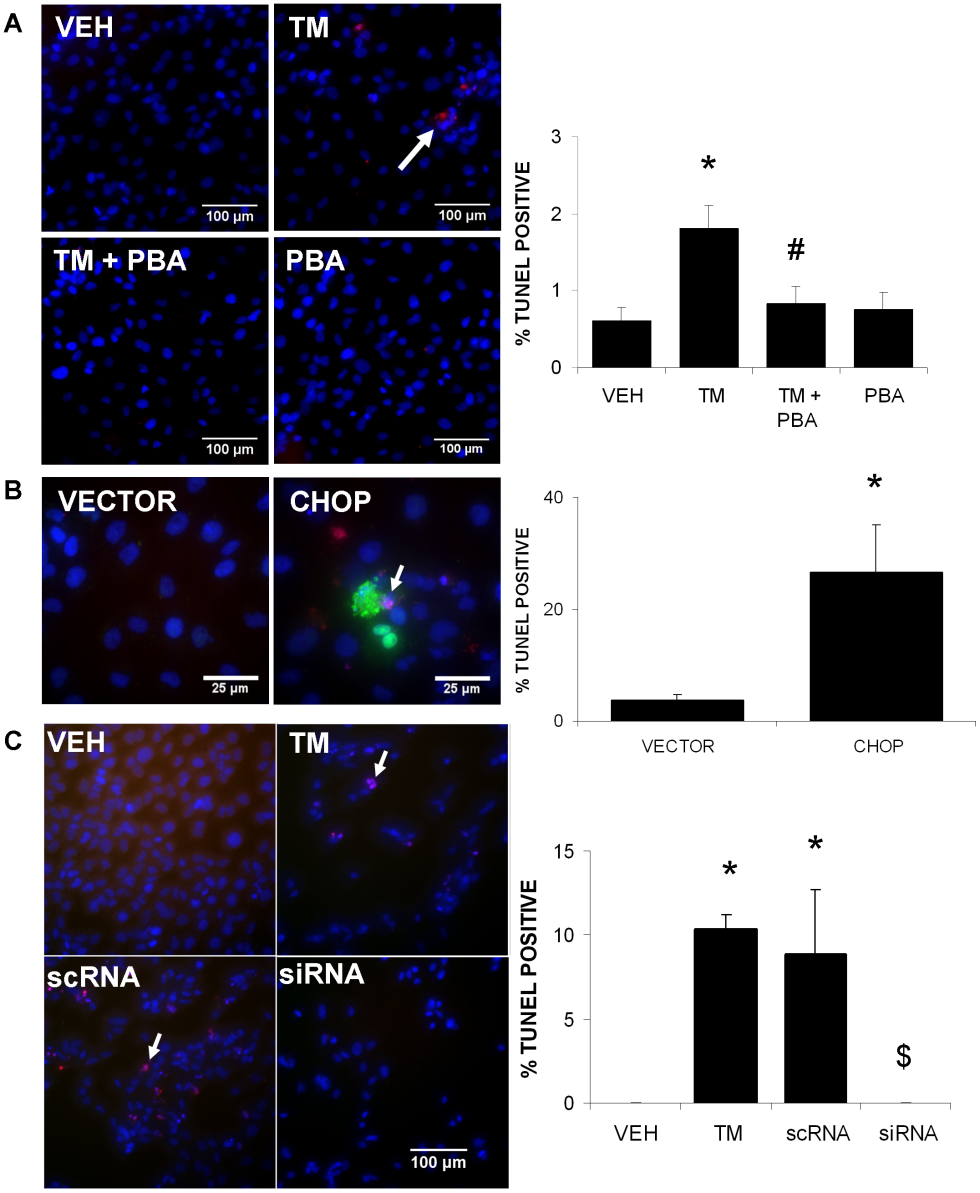
4-PBA protects against tunicamycin (TM)-induced apoptosis, which is mediated by CHOP expression. HK-2 cells were treated with DMSO (VEH), TM (1 µg/ml), TM with 4-PBA (1 mM) or 4-PBA alone for 24 h. Cells were then stained for apoptosis, using a TUNEL procedure (red). Apoptotic cells and total cells were counted. Results indicate that TM treatment significantly increased apoptosis. Co-treatment of TM with 4-PBA inhibited apoptotic cell death (A). HK-2 cells were transfected with a pcDNA3.1 control (VECTOR) or CHOP in a pcDNA3.1 vector, and immunofluorescently stained for CHOP (green) and TUNEL (red). Results indicate that increased expression of CHOP resulted in significantly more apoptosis than control-transfected cells (B). Cells were transfected with siRNA (Dharmacon ON-TARGET plus SMARTpool) to inhibit CHOP expression and then treated with TM for 48 h. Cells experienced significantly less apoptosis than non-transfected TM-treated or control-scrambled siRNA (scRNA) transfected cells (C). DAPI was used to stain all cell nuclei (blue). *, P<0.05 vs. control; ^#^, P<0.05 vs. TM; ^$^, P<0.05 vs. scRNA.

To determine if TM treatment increased apoptotic cell death in human proximal tubule cells and if 4-PBA would inhibit this effect, TUNEL staining was performed in HK-2 cells. Apoptotic cell death was measured in cells treated with VEH, TM, TM with 4-PBA, or 4-PBA alone for 24 h. Results indicate that TM treatment resulted in significantly more apoptotic cell death than VEH-treated cells. Furthermore, co-treatment with 4-PBA prevented the apoptosis (Figure 4A). To determine if CHOP overexpression alone was capable of inducing apoptosis, a pcDNA3.1 plasmid vector containing the coding region for the human CHOP protein was transfected into HK-2 cells. Cells overexpressing CHOP demonstrated significantly more apoptosis than vector control-transfected cells (Figure 4B). To determine if CHOP was responsible for the apoptosis induced by TM treatment, HK-2 cells were subjected to siRNA knockdown of CHOP, and treated with 1 μg/ml of TM for 48 h. Non-transfected cells treated with TM resulted in a significant increase in apoptosis. Cells transfected with CHOP siRNA and treated with TM showed a reduced level of apoptosis. TM-induced apoptosis was not affected by the scrambled siRNA control (scRNA; Figure 4C).

**Figure 5:**
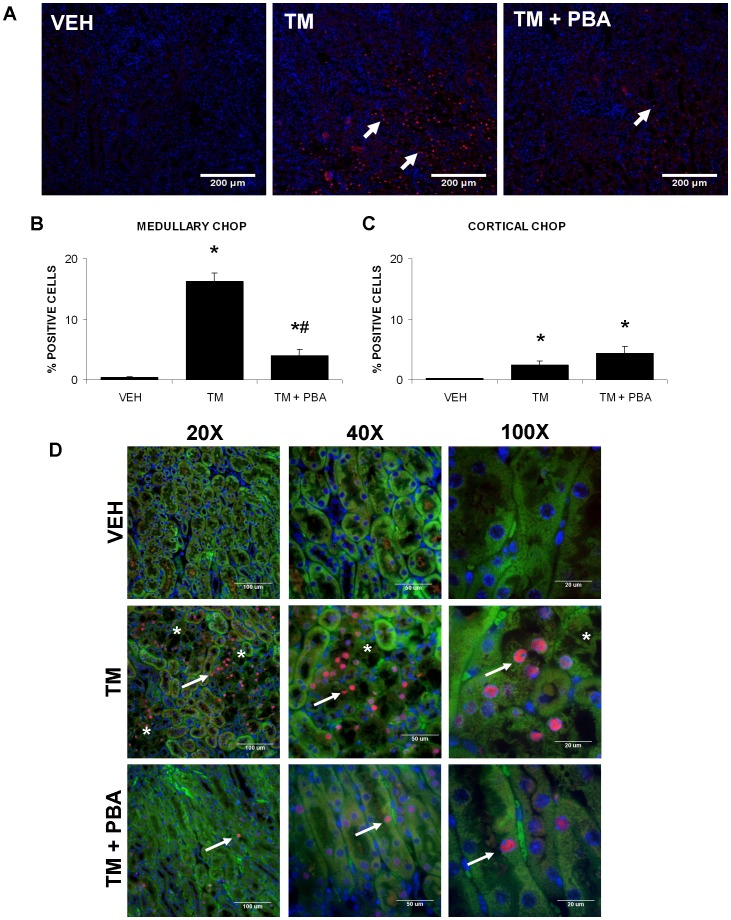
4-PBA inhibits tunicamycin (TM)-induced CHOP expression in the medulla. Mice were treated with saline (VEH), TM for 3 days, or pre-treated with 4-PBA for 7 days followed by 3 days of TM and 4-PBA co-treatment. Kidney sections from untreated or treated mice were immunofluorescently stained for CHOP (A). CHOP-stained cells and total cells were counted. Results indicate that TM upregulates CHOP expression primarily in the outer stripe of the outer medulla (arrows); TM-induced CHOP expression was lower in the cortex. Further, 4-PBA appears to attenuate the TM-induced CHOP expression in the medulla (arrows) (B), but does not have an effect in the cortex (C). Confocal images demonstrate the localization of CHOP in the nuclei of the proximal tubules (pars recta) of the kidney (arrows). *, region of damage (D). N = 9. *, P<0.05 vs VEH; ^#^, P<0.05 vs TM.

Kidney sections from the animal treatment groups were stained for CHOP (red) and its expression analyzed in both the cortex and medulla (Figure 5A). Results indicate that TM treatment induced CHOP expression in the cortex to some degree; however, CHOP expression was much greater in the renal medulla. Treatment with 4-PBA significantly inhibited TM-induced medullary CHOP expression (Figure 5B); however, it did not inhibit the lower levels of TM-induced CHOP expression in the renal cortex (Figure 5C). Kidney sections stained for CHOP were viewed in combination with 488 nm stimulated renal autofluorescence as viewed through a FITC band pass filter (green) to show renal anatomy and DAPI staining (blue) to identify nuclei. This demonstrated that the main area of CHOP staining occurred in the nuclei of proximal tubule cells of the pars recta in the outer stripe of the medulla. Confocal imaging spatially located this CHOP-linked immunofluorescent signal to the nuclei of this cell type (Figure 5D).

**Figure 6:**
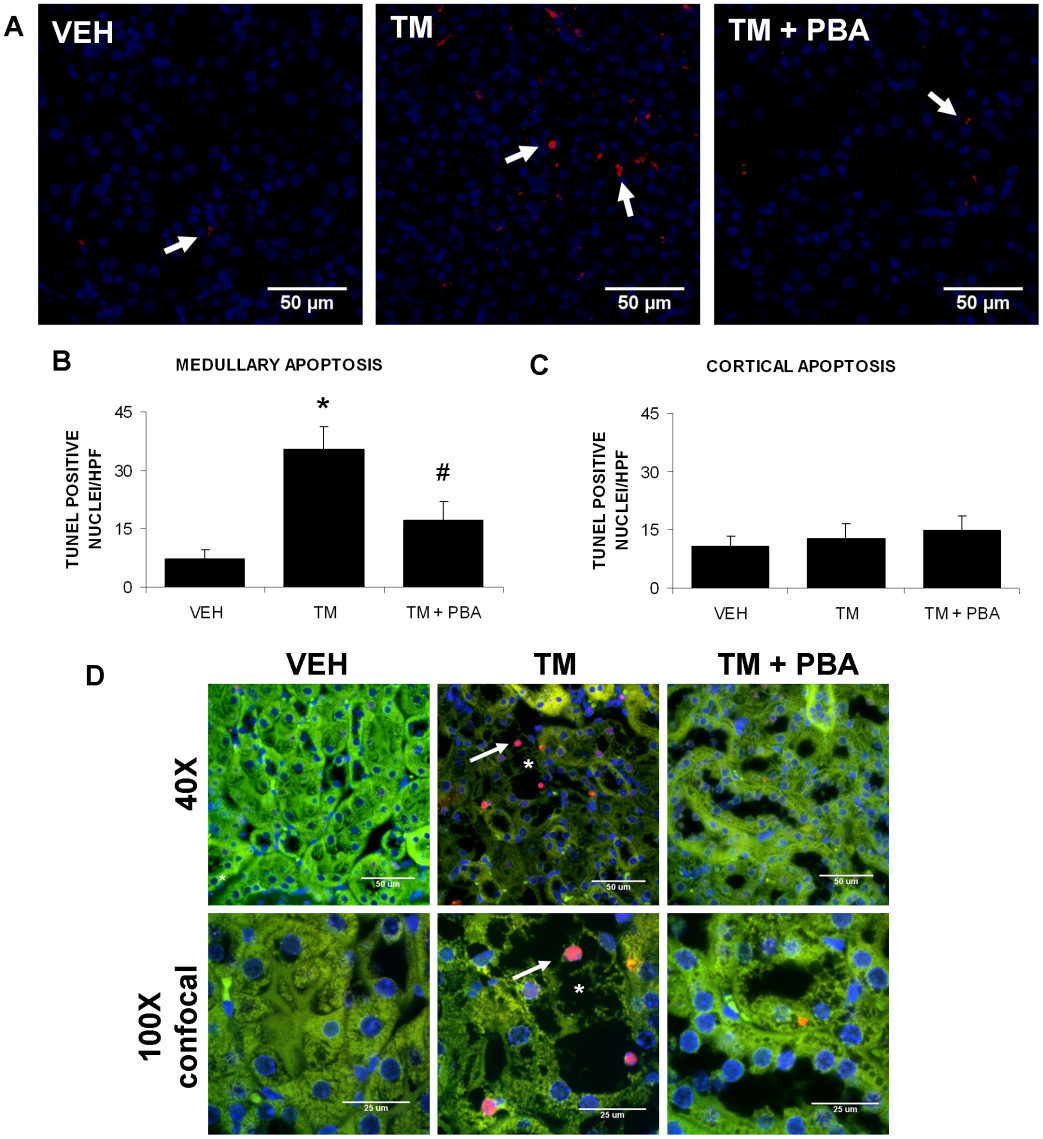
4-PBA inhibits tunicamycin (TM)-induced apoptosis in the medulla. Mice were treated with saline (VEH), TM for 3 days, or pre-treated with 4-PBA for 7 days followed by 3 days of TM and 4-PBA co-treatment. Kidney sections from untreated or treated mice were TUNEL stained for cells undergoing apoptosis (A). TUNEL-stained cells (red) were counted. Results, expressed as the number of TUNEL positive nuclei per high-powered field (HPF), indicate that TM treatment increases apoptotic cell death in the medulla (arrows). Further, 4-PBA appears to prevent the TM-induced apoptosis in the medulla (B). Apoptosis in the cortex did not significantly differ between groups (C). Confocal images demonstrated that the localization of apoptotic cells in the kidney was confined to regions of renal damage of the proximal tubular cells. *, region of damage; arrow, apoptotic nuclei (D). N = 6. *, P<0.05 vs VEH; ^#^, P<0.05 vs TM).

To determine if TM treatment increased apoptotic cell death in kidney cells *in vivo* and if 4-PBA would inhibit this effect, kidney sections were stained for apoptotic cell death with the TUNEL procedure (Figure 6A). Apoptotic cell death was measured in the renal cortex and medulla of mice treated with VEH, TM, or TM with 4-PBA. Results indicate that TM treatment resulted in significantly increased apoptotic cell death than VEH-treated cells in the renal medulla (Figure 6B) but not in the renal cortex (Figure 6C). In addition, co-treatment with 4-PBA prevented the programmed cell death in the renal medulla. Confocal images of kidney sections stained with TUNEL were viewed in combination with renal autofluorescence and DAPI staining as above, demonstrating that the main area of apoptosis occurred where renal damage occurred and the TUNEL signal originated from the nuclei of the pars recta (Figure 6D)

**Figure 7:**
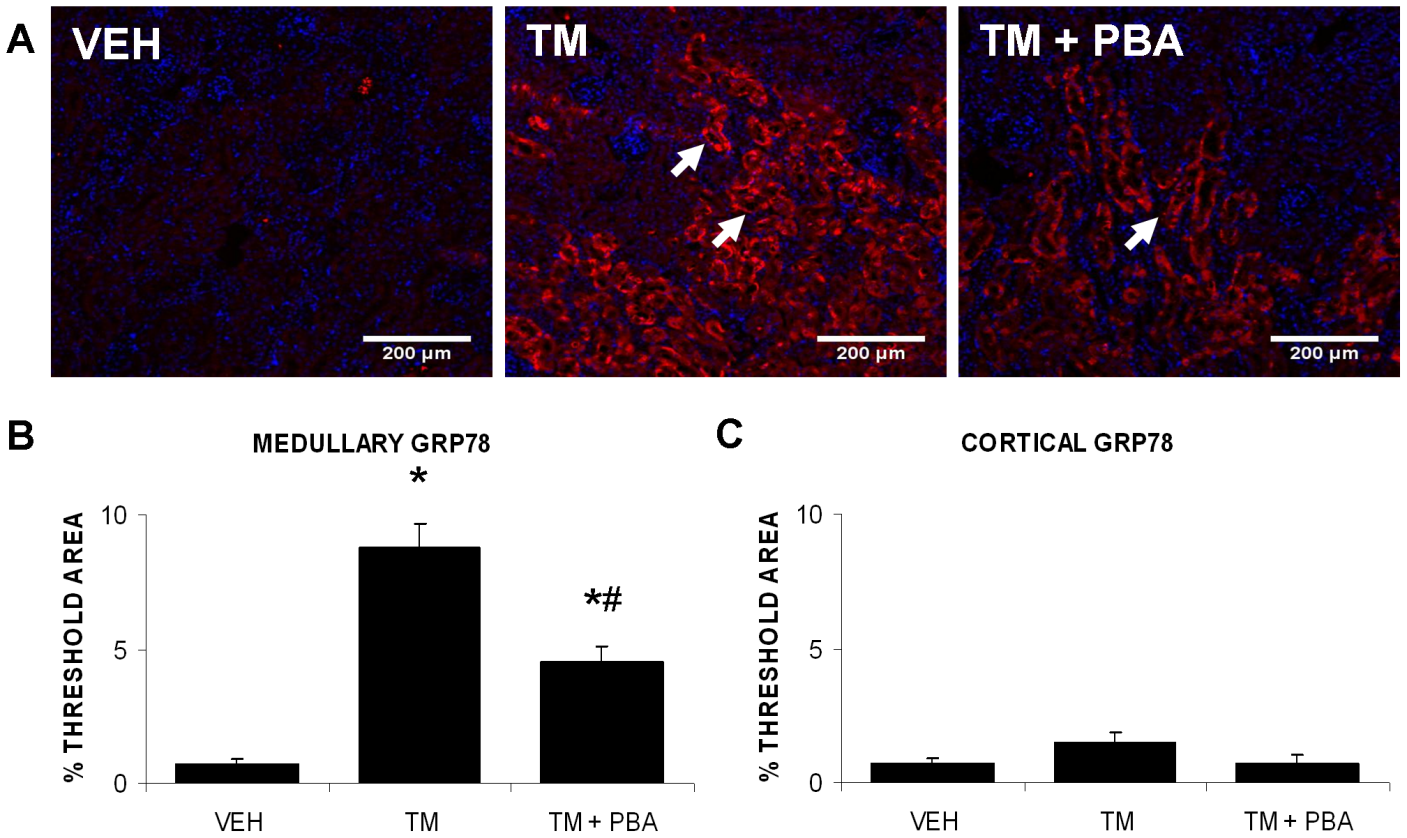
4-PBA inhibits tunicamycin (TM)-induced GRP78 expression in the medulla. Mice were treated with saline (VEH), TM for 3 days, or pre-treated with 4-PBA for 7 days followed by 3 days of TM and 4-PBA co-treatment. Kidney sections from mice were immunofluorescently stained for GRP78 (arrows) (A). The area of GRP78 staining was determined through wavelength specific thresholding. Results demonstrated that TM treatment led to upregulation of GRP78 expression, mainly in the medullary region of the kidney; this was inhibited by co-treatment with 4-PBA (B). Comparatively less GRP78 staining was found in the cortex of the kidney (C). N = 9. *, P<0.05 vs VEH; ^#^, P<0.05 vs TM.

To determine if treatment with 4-PBA affected the expression of other ER stress markers in the cortex or the medulla, kidneys were stained for GRP78 (Figure 7A). Analysis demonstrated that GRP78 expression was significantly increased in the medulla of TM-treated kidneys; this effect was inhibited by 4-PBA (Figure 7B). GRP78 expression was not increased in the cortex (Figure 7C).

**Figure 8:**
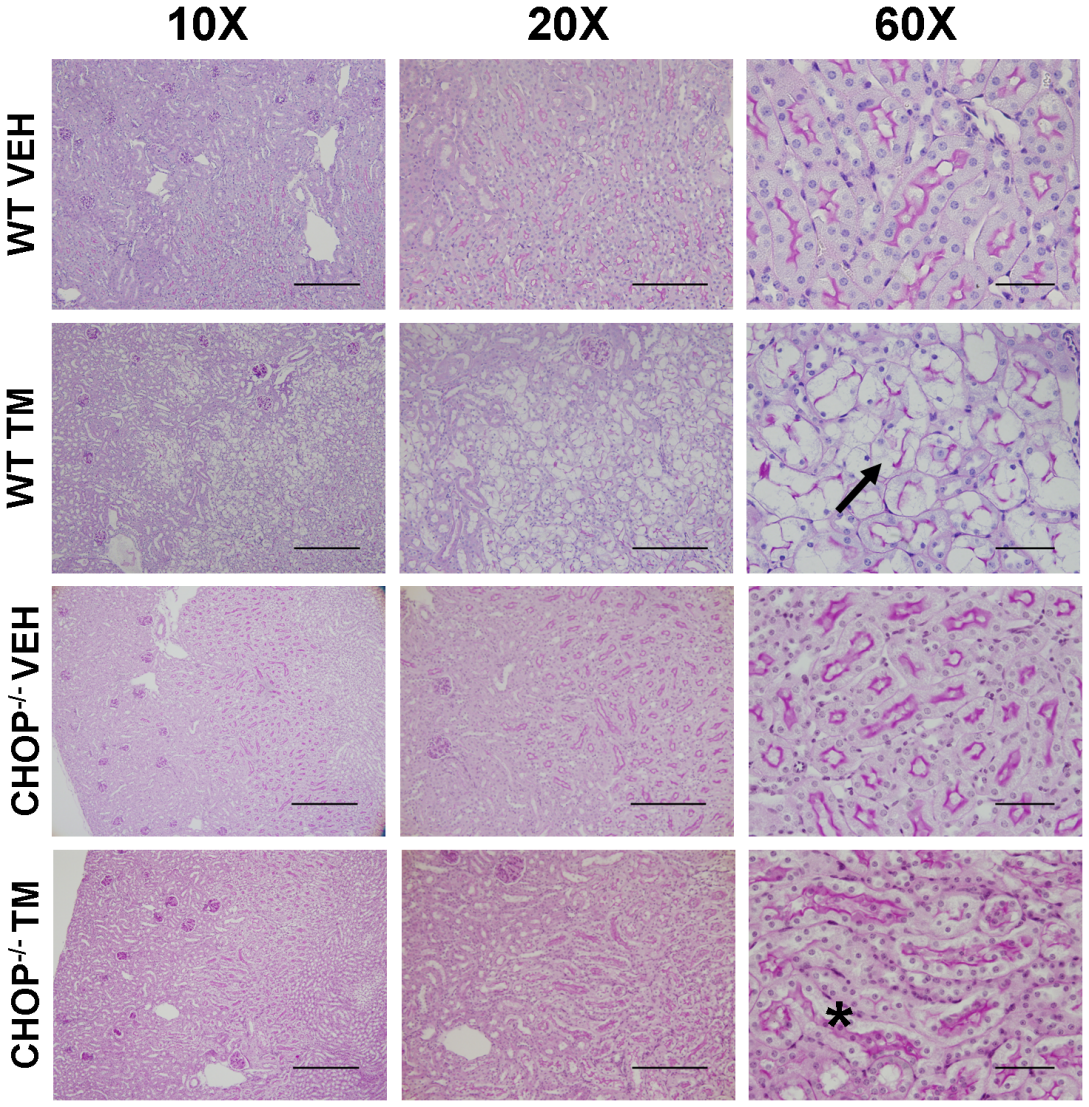
CHOP deficiency protects against tunicamycin (TM)-induced acute kidney injury. Kidney sections from C57BL/6 wild type mice and CHOP^−/−^ mice treated with saline (VEH) or TM for 3 days were PAS stained. Staining demonstrates that CHOP^−/−^ mice treated with TM did not display pathological signs of acute kidney injury like wild type mice, including those found in the pars recta (*). High magnification images are of the outer stripe of the outer medulla, the main area of damage in the wild type mice (arrow). 10×, bar = 350 µm; 20×, bar = 200 µm; 60×, bar = 50 µm.

To investigate whether increased CHOP expression alone may be responsible for the AKI induced by our TM model and whether inhibiting CHOP expression could explain 4-PBA's protective effect, CHOP^−/−^ mice were subjected to the TM model. PAS staining was used to examine kidney damage in wild type and CHOP^−/−^ mouse kidneys. As previously described, wild type TM-treated mouse kidneys suffered from tubular atrophy, loss of brush border, and epithelial cell vacuolization in the outer stripe of the outer medulla (arrow). However, CHOP^−/−^ mice treated with TM did not suffer from any noticeable kidney damage and showed intact pars recta (*) (Figure 8). Blood plasma was analyzed for serum creatinine, and it was determined that creatinine levels from TM-treated CHOP^−/−^ mice did not differ from levels from wild type mice (wild type, 34.5±2.23 vs CHOP^−/−^, 34.8±1.19 μmol/L), both of which were within the normal range.

**Figure 9:**
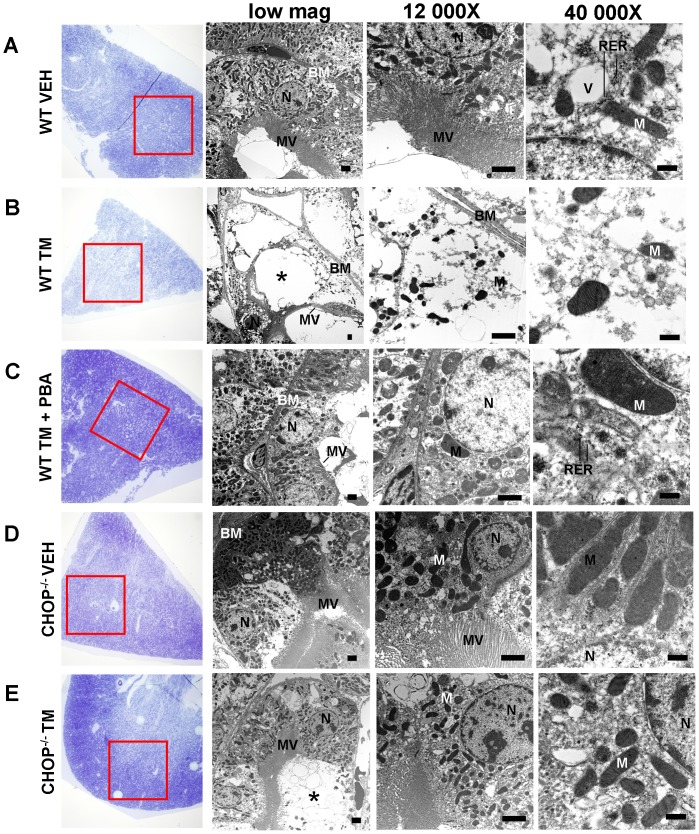
4-PBA protects ultrastructure of pars recta against tunicamycin (TM)-induced acute kidney injury. Transmission electron microscopy was used to visualize the ultrastructure of proximal tubule epithelial cells in the pars recta of kidneys from wild type and CHOP^−/−^ mice. Low magnification image (blue sections) show regions of outer medulla prepared for ultra-thin sectioning (red box). Proximal tubule cells of wild type sham-treated mice within this region showed prominent brush border with microvilli and nuclei. Detailed images show numerous mitochondria in the perinuclear region, rough endoplasmic reticulum and pinocytotic vesicles (A). Wild type mice treated with TM (0.5 mg/kg) showed severe disruption of proximal tubule cells within the outer medulla where most of the cytoplasm of these cells was lost or degraded (*). However, microvilli on the brush border remained visible, as well as nuclei and mitochondria within the cytoplasm. Further, the basement membrane remained intact (B). In contrast, co-treatment of TM with 4-PBA (1 g/kg/day) preserved the proximal tubule cells of the outer stripe, which displayed intact cytoplasm, undamaged nuclei, mitochondria, and rough endoplasmic reticulum (C). Kidneys of CHOP^−/−^ mice displayed proximal tubule cells in the outer medulla, which did not differ from those of wild type mice (D). CHOP deficiency protected the proximal tubular cells from TM treatment, maintaining undamaged nuclei, mitochondria and rough endoplasmic reticulum in the cytoplasm. Infrequent cells displaying ultrastructural degeneration similar to that found in wild type TM-treated mice remained in TM-treated CHOP^−/−^ mice (*) (E). MV, microvilli; N, nuclei; M, mitochondria; RER, rough endoplasmic reticulum; V, pinocytotic vesicles; BM, basement membrane. Low mag, bar = 2 µm; 12 000×, bar = 2 µm; 40 000×, bar = 500 nm.

To determine if 4-PBA preserved the ultrastructural integrity of proximal tubule cells with TM treatment, we utilized TEM. Care was taken to focus on the outer medulllary stripe. Wild type TM-treated mice demonstrated severe ultrastructural injury in proximal tubule cells. Examination of a number of cellular organelles, including nuclei, mitochondria, rough ER, as well as microvilli, revealed that 4-PBA inhibited TM-induced ultrastructural injury. Further, the ultrastructure of TM-treated CHOP^−/−^ mice appeared similar to that of VEH-treated wild type and CHOP^−/−^ mice and showed less ultrastructural abnormalities than TM-treated wild type mice (Figure 9).

## Discussion

TM is a well-known ER stress inducer, and has previously been used as a model of antibiotic-induced AKI [11], [12]. Using mouse embryonic fibroblasts, researchers demonstrated that knockout of CHOP inhibits TM-induced effects, including decreased levels of GADD34 expression, and increased phosphorylation of eIF2α. CHOP knockout also prevents TM-induced AKI in mice. CHOP heterozygous mice treated with TM suffered from pyknotic nuclei (a marker of apoptosis), cellular casts in the urine, and cellular debris in the tubules, unlike CHOP^−\−^ mice. CHOP^−\−^ mice also displayed significantly less apoptosis. Additionally, in mice with mutant GADD34 incompetent to bind to PP1 and dephosphorylate eIF2α, protection against TM-induced AKI is similarly observed [5]. This model of ER stress-induced AKI differed from our model only in the dose of TM applied, where previously 1.0 mg/kg was used, we used a lower dose of 0.5 mg/kg. Our results in regard to the protective effect of CHOP knockout at this lower dose of AKI induction are in agreement with the previous study.

We found that 4-PBA was able to prevent or partially prevent the pathologic changes associated with TM-induced AKI. As previously mentioned, 4-PBA has an effect as an ER stress inhibitor [18]–[20]; however, 4-PBA also acts as a HDAC inhibitor [17]. Its effects on protein acetylation may extend beyond those affecting histones. HDAC inhibitors are known to prevent deacetylation of spliced XBP1, increasing stability and transcriptional activity [33]. Spliced XBP1 is a key transcriptional inducer of the IRE1 arm of the UPR and may protect against apoptosis by upregulating the expression of protein folding chaperones, including GRP78 [34]. If 4-PBA increased spliced XBP1 stability and transcriptional activity, it may have led to increased GRP78 expression early in TM-induced AKI (18–24 hours). This would have resulted in reduced UPR activation through GRP78-mediated repression of PERK and IRE1 autophosphorylation. Diminished PERK response would then have resulted in the lower TM-induced CHOP expression observed at the 3-day time point. However, we found a repression of GRP78 expression at the 3-day time point in our TM model, coinciding with CHOP repression. This suggests that the primary mode of action for 4-PBA, in the animal model, was to repress ER stress in general and protect against renal epithelial cell death unrelated to its HDAC inhibitor properties. A similar effect of 4-PBA treatment on the inhibition of TM-induced neuronal cell death was attributed to be due to its protein folding chaperone properties, rather than its HDAC inhibitor properties through the derivation of compounds with similar molecular structure but varying protein folding chaperone and HDAC inhibitor properties [35].

4-PBA has previously been shown to repress ER stress. Liver and adipose tissue of *ob/ob* mice treated with 4-PBA displayed significantly lower levels of the ER stress markers, phospho-PERK and phospho-IRE1, when compared with vehicle-treated mice [21]. Similar results were found *in vitro*, with reduced levels of GRP78 and phospho-eIF2α found in rat hepatoma cells treated with 4-PBA [36]. In fact, 4-PBA may even alleviate lipid-induced insulin resistance and β-cell dysfunction in obese individuals by inhibiting ER stress [19]. The main effect of 4-PBA treatment on human proximal tubular cells *in vitro* was to repress the pro-apoptotic gene CHOP induced by TM treatment while maintaining cytoprotective levels of GRP78 expression. Further, we demonstrated through plasmid-mediated CHOP expression that CHOP overexpression alone was capable of inducing proximal tubular cell apoptosis and that the siRNA-mediated inhibition of CHOP expression prevented apoptosis.

In the animal model, TM-induced AKI resulted in increased GRP78 and CHOP staining in mouse kidneys, indicating the development of ER stress. As previously noted, GRP78 is a common marker of ER stress, as it binds to unfolded or misfolded proteins in the ER and is transcriptionally upregulated by ER stress through the UPR [1]. CHOP is a transcription factor involved in ER stress, and has been characterized as a pro-apoptotic protein [5]. Treatment with TM increased levels of ER stress markers, such as CHOP, particularly in the pars recta of the renal medulla. Others have demonstrated intense CHOP nuclear staining in the cortex of kidneys from TM-treated mice [11]. We suspect this variation is due to different concentrations of TM treatment, with higher concentrations causing more severe renal damage in the cortex. As previously mentioned, we treated our mice with 0.5 mg/kg TM, while researchers demonstrating cortical CHOP staining utilized a higher dose of 1.0 mg/kg TM [11]. Treatment with 4-PBA resulted in significantly lower levels of ER stress marker proteins, demonstrating inhibition of ER stress in the kidney with 4-PBA treatment. These results indicate that the major mechanism by which 4-PBA prevents TM-induced AKI may be through inhibition of ER stress. This inhibition of ER stress in the kidneys also coincided with 4-PBA's inhibition of renal apoptosis in the pars recta.

Through our TEM examination, we found that treatment with 4-PBA prevented TM-mediated ultrastructural damage to the proximal tubule cells in the pars recta of the outer medulla. We found through ultrastructural examination that CHOP knock out had a protective effect against TM, similar to treatment with 4-PBA. We suggested previously that 4-PBA prevents TM-induced kidney damage through the inhibition of ER stress. Since we have demonstrated that 4-PBA inhibits the expression of CHOP induced by TM and that CHOP^−/−^ mice are also protected at the ultrastructural level from TM-induced toxicity, we conclude that 4-PBA prevents TM-induced kidney damage through a CHOP-mediated mechanism.

As such, the ability of 4-PBA to directly aid in the reduction of misfolded proteins in the ER [37], [38] appears to be the molecular mechanism behind its ability to inhibit ER stress, as shown by repressed UPR marker expression of both GRP78 and CHOP *in vivo*. In particular, the reduction in CHOP expression may be responsible for preventing the tubular damage associated with TM-induced nephrotoxicity. This conclusion is corroborated by previous reports demonstrating similar results, in which CHOP^−\−^ and GADD34 mutant mice were protected from TM-induced kidney damage. In these studies, wild type mice suffered from extensive tubular interstitial damage, including apoptosis at the cortico-medullary junction [5], similar to ATN. A further study demonstrated that TRIF-dependent toll-like receptor engagement, brought about by lipopolysaccharide treatment, repressed CHOP expression and renal injury in TM-treated mice [39]. As mentioned, these TM-induced insults are comparable to our results, suggesting that ER stress is the main mechanism by which TM causes AKI. This also indicates that it is by inhibiting ER stress, and in particular CHOP expression, that 4-PBA protects the kidney from the damage caused by TM. To confirm these findings we also subjected CHOP^−/−^ mice to our model of TM-induced AKI and demonstrated that CHOP deletion in the presence of TM treatment prevented ER stress-induced AKI, at both a light level and ultrastructurally. Thus, 4-PBA, being an approved pharmaceutical with demonstrated CHOP repressing action, becomes a practical technique to modulate CHOP expression.

## Conclusions

4-PBA prevents TM-induced AKI in C57BL/6 mice. The main mechanism of 4-PBA's inhibitory effect appears to be the repression of CHOP expression *in vitro* in human proximal tubular cells and *in vivo* in the proximal tubule of the outer stripe of the outer medulla. 4-PBA's inhibition of CHOP expression is correlated with its ability to inhibit TM-induced apoptosis. Thus, our results support the hypothesis that 4-PBA exerts its protective effects on TM-induced AKI by inhibiting CHOP expression through the inhibition of ER stress. Given the fact that 4-PBA is in current clinical use and is well-tolerated, it is an attractive agent for study in situations where AKI might be expected to occur.

## References

[1] Schroder M, Kaufman RJ (2005) ER stress and the unfolded protein response. Mutat Res 569: 29–63. 15603751 10.1016/j.mrfmmm.2004.06.056

[2] Dickhout JG, Carlisle RE, Austin RC (2011) Inter-Relationship between Cardiac Hypertrophy, Heart Failure and Chronic Kidney Disease – Endoplasmic Reticulum Stress as a Mediator of Pathogenesis. Circ Res 108: 629–642. 21372294 10.1161/CIRCRESAHA.110.226803

[3] Dickhout JG, Krepinsky JC (2009) Endoplasmic reticulum stress and renal disease. Antioxid Redox Signal 11: 2341–2352. 19508129 10.1089/ars.2009.2705

[4] Walter P, Ron D (2011) The unfolded protein response: from stress pathway to homeostatic regulation. Science 334: 1081–1086. 22116877 10.1126/science.1209038

[5] Marciniak SJ, Yun CY, Oyadomari S, Novoa I, Zhang Y, et al (2004) CHOP induces death by promoting protein synthesis and oxidation in the stressed endoplasmic reticulum. Genes Dev 18: 3066–3077. 15601821 10.1101/gad.1250704 PMC535917

[6] Prachasilchai W, Sonoda H, Yokota-Ikeda N, Ito K, Kudo T, et al (2009) The protective effect of a newly developed molecular chaperone-inducer against mouse ischemic acute kidney injury. J Pharmacol Sci 109: 311–314. 19179808 10.1254/jphs.08272sc

[7] Prachasilchai W, Sonoda H, Yokota-Ikeda N, Oshikawa S, Aikawa C, et al (2008) A protective role of unfolded protein response in mouse ischemic acute kidney injury. Eur J Pharmacol 592: 138–145. 18644364 10.1016/j.ejphar.2008.06.108

[8] Peyrou M, Hanna PE, Cribb AE (2007) Calpain inhibition but not reticulum endoplasmic stress preconditioning protects rat kidneys from p-aminophenol toxicity. Toxicol Sci 99: 338–345. 17567592 10.1093/toxsci/kfm105

[9] Peyrou M, Cribb AE (2007) Effect of endoplasmic reticulum stress preconditioning on cytotoxicity of clinically relevant nephrotoxins in renal cell lines. Toxicol In Vitro 21: 878–886. 17416481 10.1016/j.tiv.2007.03.001

[10] Wu CT, Sheu ML, Tsai KS, Weng TI, Chiang CK, et al (2010) The role of endoplasmic reticulum stress-related unfolded protein response in the radiocontrast medium-induced renal tubular cell injury. Toxicol Sci 114: 295–301. 20071420 10.1093/toxsci/kfq006

[11] Zinszner H, Kuroda M, Wang X, Batchvarova N, Lightfoot RT, et al (1998) CHOP is implicated in programmed cell death in response to impaired function of the endoplasmic reticulum. Genes Dev 12: 982–995. 9531536 10.1101/gad.12.7.982 PMC316680

[12] Lhotak S, Sood S, Brimble E, Carlisle RE, Colgan SM, et al (2012) ER stress contributes to renal proximal tubule injury by increasing SREBP-2-mediated lipid accumulation and apoptotic cell death. Am J Physiol Renal Physiol 303: F266–278. 22573382 10.1152/ajprenal.00482.2011

[13] Yan K, Khoshnoodi J, Ruotsalainen V, Tryggvason K (2002) N-linked glycosylation is critical for the plasma membrane localization of nephrin. J Am Soc Nephrol 13: 1385–1389. 11961028 10.1097/01.asn.0000013297.11876.5b

[14] Nakagawa T, Zhu H, Morishima N, Li E, Xu J, et al (2000) Caspase-12 mediates endoplasmic-reticulum-specific apoptosis and cytotoxicity by amyloid-beta. Nature 403: 98–103. 10638761 10.1038/47513

[15] Huang L, Zhang R, Wu J, Chen J, Grosjean F, et al (2011) Increased susceptibility to acute kidney injury due to endoplasmic reticulum stress in mice lacking tumor necrosis factor-alpha and its receptor 1. Kidney Int 79: 613–623. 21150875 10.1038/ki.2010.469

[16] Lichter-Konecki U, Diaz GA, Merritt JL 2nd, Feigenbaum A, Jomphe C, et al (2011) Ammonia control in children with urea cycle disorders (UCDs); phase 2 comparison of sodium phenylbutyrate and glycerol phenylbutyrate. Mol Genet Metab 103: 323–329. 21612962 10.1016/j.ymgme.2011.04.013 PMC4880058

[17] Miller AC, Cohen S, Stewart M, Rivas R, Lison P (2011) Radioprotection by the histone deacetylase inhibitor phenylbutyrate. Radiat Environ Biophys 50: 585–596. 21892632 10.1007/s00411-011-0384-7

[18] Basseri S, Lhotak S, Sharma AM, Austin RC (2009) The chemical chaperone 4-phenylbutyrate inhibits adipogenesis by modulating the unfolded protein response. J Lipid Res 50: 2486–2501. 19461119 10.1194/jlr.M900216-JLR200 PMC2781320

[19] Xiao C, Giacca A, Lewis GF (2011) Sodium phenylbutyrate, a drug with known capacity to reduce endoplasmic reticulum stress, partially alleviates lipid-induced insulin resistance and beta-cell dysfunction in humans. Diabetes 60: 918–924. 21270237 10.2337/db10-1433 PMC3046853

[20] Yam GH, Gaplovska-Kysela K, Zuber C, Roth J, Yam GH-F, et al (2007) Sodium 4-phenylbutyrate acts as a chemical chaperone on misfolded myocilin to rescue cells from endoplasmic reticulum stress and apoptosis. Investigative Ophthalmology & Visual Science 48: 1683–1690. 17389500 10.1167/iovs.06-0943

[21] Ozcan U, Yilmaz E, Ozcan L, Furuhashi M, Vaillancourt E, et al (2006) Chemical chaperones reduce ER stress and restore glucose homeostasis in a mouse model of type 2 diabetes. Science 313: 1137–1140. 16931765 10.1126/science.1128294 PMC4741373

[22] Loffing J, Moyer BD, Reynolds D, Stanton BA (1999) PBA increases CFTR expression but at high doses inhibits Cl(-) secretion in Calu-3 airway epithelial cells. Am J Physiol 277: L700–708. 10516210 10.1152/ajplung.1999.277.4.L700

[23] Collins AF, Pearson HA, Giardina P, McDonagh KT, Brusilow SW, et al (1995) Oral sodium phenylbutyrate therapy in homozygous beta thalassemia: a clinical trial. Blood 85: 43–49. 7528572

[24] Mimori S, Okuma Y, Kaneko M, Kawada K, Hosoi T, et al (2012) Protective effects of 4-phenylbutyrate derivatives on the neuronal cell death and endoplasmic reticulum stress. Biol Pharm Bull 35: 84–90. 22223342 10.1248/bpb.35.84

[25] Dyer ES, Paulsen MT, Markwart SM, Goh M, Livant DL, et al (2002) Phenylbutyrate inhibits the invasive properties of prostate and breast cancer cell lines in the sea urchin embryo basement membrane invasion assay. Int J Cancer 101: 496–499. 12216080 10.1002/ijc.10609

[26] Carducci MA, Nelson JB, Chan-Tack KM, Ayyagari SR, Sweatt WH, et al (1996) Phenylbutyrate induces apoptosis in human prostate cancer and is more potent than phenylacetate. Clin Cancer Res 2: 379–387. 9816181

[27] Phuphanich S, Baker SD, Grossman SA, Carson KA, Gilbert MR, et al (2005) Oral sodium phenylbutyrate in patients with recurrent malignant gliomas: a dose escalation and pharmacologic study. Neuro Oncol 7: 177–182. 15831235 10.1215/S1152851704000183 PMC1871887

[28] Ryan MJ, Johnson G, Kirk J, Fuerstenberg SM, Zager RA, et al (1994) HK-2: an immortalized proximal tubule epithelial cell line from normal adult human kidney. Kidney Int 45: 48–57. 8127021 10.1038/ki.1994.6

[29] Hossain GS, van Thienen JV, Werstuck GH, Zhou J, Sood SK, et al (2003) TDAG51 is induced by homocysteine, promotes detachment-mediated programmed cell death, and contributes to the development of atherosclerosis in hyperhomocysteinemia. J Biol Chem 278: 30317–30327. 12738777 10.1074/jbc.M212897200

[30] Dickhout JG, Lhotak S, Hilditch BA, Basseri S, Colgan SM, et al (2011) Induction of the unfolded protein response after monocyte to macrophage differentiation augments cell survival in early atherosclerotic lesions. FASEB J 25: 576–589. 20966213 10.1096/fj.10-159319

[31] Dickhout JG, Carlisle RE, Jerome DE, Mohammed-Ali Z, Jiang H, et al (2012) Integrated Stress Response Modulates Cellular Redox State via Induction of Cystathionine gamma-Lyase: CROSS-TALK BETWEEN INTEGRATED STRESS RESPONSE AND THIOL METABOLISM. J Biol Chem 287: 7603–7614. 22215680 10.1074/jbc.M111.304576 PMC3293561

[32] Carlisle RE, Heffernan A, Brimble E, Liu L, Jerome D, et al (2012) TDAG51 mediates epithelial-to-mesenchymal transition in human proximal tubular epithelium. Am J Physiol Renal Physiol 303: F467–481. 22592641 10.1152/ajprenal.00481.2011

[33] Wang FM, Chen YJ, Ouyang HJ (2011) Regulation of unfolded protein response modulator XBP1s by acetylation and deacetylation. Biochem J 433: 245–252. 20955178 10.1042/BJ20101293 PMC3477812

[34] Hosoi T, Korematsu K, Horie N, Suezawa T, Okuma Y, et al (2012) Inhibition of casein kinase 2 modulates XBP1-GRP78 arm of unfolded protein responses in cultured glial cells. PLoS One 7: e40144. 22768244 10.1371/journal.pone.0040144 PMC3387139

[35] Mimori S, Ohtaka H, Koshikawa Y, Kawada K, Kaneko M, et al (2013) 4-Phenylbutyric acid protects against neuronal cell death by primarily acting as a chemical chaperone rather than histone deacetylase inhibitor. Bioorg Med Chem Lett 23: 6015–6018. 24044874 10.1016/j.bmcl.2013.08.001

[36] Ota T, Gayet C, Ginsberg HN (2008) Inhibition of apolipoprotein B100 secretion by lipid-induced hepatic endoplasmic reticulum stress in rodents. J Clin Invest 118: 316–332. 18060040 10.1172/JCI32752 PMC2104481

[37] de Almeida SF, Picarote G, Fleming JV, Carmo-Fonseca M, Azevedo JE, et al (2007) Chemical chaperones reduce endoplasmic reticulum stress and prevent mutant HFE aggregate formation. J Biol Chem 282: 27905–27912. 17626021 10.1074/jbc.M702672200

[38] Wang WJ, Mulugeta S, Russo SJ, Beers MF (2003) Deletion of exon 4 from human surfactant protein C results in aggresome formation and generation of a dominant negative. J Cell Sci 116: 683–692. 12538769 10.1242/jcs.00267

[39] Woo CW, Cui D, Arellano J, Dorweiler B, Harding H, et al (2009) Adaptive suppression of the ATF4-CHOP branch of the unfolded protein response by toll-like receptor signalling. Nat Cell Biol 11: 1473–1480. 19855386 10.1038/ncb1996 PMC2787632

